# Screening Bleeding Disorders in Adolescents and Young Women with Menorrhagia

**DOI:** 10.4274/Tjh.2011.0048

**Published:** 2013-06-05

**Authors:** Suar Çakı Kılıç, Nazan Sarper, Emine Zengin, Sema Aylan Gelen

**Affiliations:** 1 Kocaeli University Medical Faculty, Department of Pediatric Hematology, Kocaeli, Turkey

**Keywords:** Menorrhagia, Bleeding disorder, von Willebrand disease, Platelet aggregation defects

## Abstract

**Objective:** Chronic menorrhagia causes anemia and impairment of life quality. In this study the aim was the screening of bleeding disorders in adolescents and young women with menorrhagia.

**Materials and Methods: **The study was performed prospectively by pediatric hematologists. A form including demographic characteristics of the patients, bleedings other than menorrhagia, familial bleeding history, characteristics of the menorrhagia, and impairment of life quality due to menorrhagia was filled out by the researcher during a face-to-face interview with the patient. A pictorial blood assessment chart was also used for evaluation of blood loss. All patients underwent pelvic ultrasound sonography testing and women also received pelvic examination by gynecologists. Whole blood count, peripheral blood smear, blood group, serum transaminases, urea, creatinine, ferritin, PFA-100, PT, aPTT, INR, TT, fibrinogen, VWF:Ag, VWF:RCo, FVIII, and platelet aggregation assays were performed. Platelet aggregations were studied by lumiaggregometer.

**Results:** Out of 75 patients enrolled, 60 patients completed the study. The mean age was 20.68±10.34 (range: 10-48) years and 65% (n=39) of the patients were younger than 18 years. In 18 (46%) of the adolescents, menorrhagia subsided spontaneously. In 20% (n=12) of the patients, a bleeding disorder was detected (1 case of type 3 von Willebrand disease, 2 patients with low VWF:Ag, 1 case of probable von Willebrand disease, 3 cases of Bernard-Soulier syndrome, 2 cases of Glanzmann thrombasthenia, 2 cases of immune thrombocytopenic purpura, 1 case of congenital factor VII deficiency).

**Conclusion:** In patients with menorrhagia, at least complete blood count, peripheral smear, aPTT, PT, VWF:Ag, VWF:RCo, FVIII, and fibrinogen assays must be performed. When there is history of nose and gum bleeding, platelet function assay by lumiaggregometer must also be performed. In nearly 50% of adolescents, menorrhagia is dysfunctional and transient. Detailed coagulation assays can be postponed in adolescents if bleeding history other than menorrhagia and/or family history of bleeding and/or parental consanguinity is absent. All subjects with menorrhagia must consult with gynecologists and hematologists.

**Conflict of interest:**None declared.

## Abstract (tr)

**Amaç:** Kronik menoraji anemiye ve yaşam kalitesinde bozulmaya neden olur. Bu çalışmanın amacı adolesan ve genç kadınlarda pıhtılaşma bozukluklarının taranmasıdır.

**Gereç ve Yöntemler: **Bu çalışma çocuk hematoloji hekimleri tarafından prospektif olarak yapıldı. Araştıcı tarafından hastayla karşılıklı yapılan görüşmede hastaların demografik özelliklerini, menoraji dışındaki diğer kanama öyküsünü, aile öyküsünü, yoğun menstruasyon kanamasının özelliklerini ve bu durumun yaşam kalitesini etkileme derecesini içeren bir form dolduruldu. Menstruasyondaki kan kaybının yoğunluğunu değerlendirmek için resimli kanama değerlendirme çizelgesi kullanıldı. Tüm hastalara jinekologlar tarafından pelvik muayene ve ultrasonografi yapıldı. Tam kan sayımı, periferik yayma, kan grubu, serum transaminazları, üre, kreatinin, ferritin, PFA-100, PZ, aPTZ, INR, TT, fibrinojen, VWF: Ag, VWF: RCo, FVIII, trombosit agregasyon testleri yapıldı. Trombosit agregasyon testleri lumiagregometri yöntemiyle çalışıldı.

**Bulgular:** Çalışmaya 75 hasta ile başlandı 60 hasta çalışmayı tamamladı. Ortalama yaş 20,68±10,34 (10-48) olan hastaların %65’i (n=39) 18 yaşın altındaydı. Adolesanların %46’sında (n=18) menoraji kendiliğinden düzeldi. Hastaların %20’sinde (n=12) pıhtılaşma bozukluğu saptandı. (1 hasta Tip III Von Willebrand hastalığı, 2 hasta düşük VWF: Ag, 1 hasta olası Von Willebrand hastalığı, 3 hasta Bernard-Soulier sendromu, 2 hasta Glanzmann trombastenisi, 2 hasta immun trombositopenik purpura, 1 hasta doğumsal FVII eksikliği).

**Sonuç:** Menorajili hastalarda başlangıçta tam kan sayımı, periferik yayma, aPTZ, PZ, VWF:Ag, VWF:RCo, FVIII ve fibrinojen düzeyleri bakılmalıdır. Burun ve dişeti kanaması hikayesi varlığında, trombosit fonksiyonları lumiagregometrik yöntemle değerlendirilmelidir. Adolesanların yaklaşık %50’sinde menoraji hormonal fonksiyon bozukluğuna bağlı ve geçicidir. Detaylı pıhtılaşma testlerinin yapılması menoraji dışında kanama hikayesi olanlar ve/veya ailevi kanama hikayesi olanlar ve/veya akraba evliliği olan hastalar dışında ertelenmelidir. Tüm menorajili hastalar jinekoloji ve hematoloji hekimleri tarafından değerlendirilmelidir.

## INTRODUCTION

Menorrhagia is defined as a menstrual blood loss of more than 80 mL per menstrual cycle. Its estimated prevalence in healthy women is 9%-14% [1]. A variety of organic, endocrine, gynecologic, or other systemic causes may be responsible for menorrhagia [2]. Menstrual problems are likely to be worse in women with bleeding disorders, as they are more likely to have heavy and painful menstrual periods and ovulation bleeding and pain [3,4]. The excessive blood loss can cause anemia and tiredness. In various published series, 32%-100% of women with von Willebrand disease (VWD), the most common inherited bleeding disorder, were reported to have heavy menstrual bleeding. Heavy menstrual bleedings were reported among 10%-70% of women with other bleeding disorders [5]. Underlying bleeding disorders were generally missed due to unavailability of detailed coagulation assays in routine laboratory procedures and lack of hematology consultation. The aim of this study was the screening of bleeding disorders in adolescents and young women presenting with menorrhagia. 

## MATERIALS AND METHODS

The ethics committee of Kocaeli University approved the study. The patients’, and for patients younger than 18 years also their legal guardians’, written informed consent was obtained. The study was performed prospectively by pediatric hematologists between June 2009 and December 2010 in adolescents and women of reproductive age during their admission to the gynecology and adult and pediatric hematology outpatient and emergency care units of Kocaeli University Hospital. Physicians of the Gynecology Department were informed about the study and referrals of patients presenting with menorrhagia were requested. The adult hematology unit was also informed about the study and referral of patients presenting with menorrhagia was requested. Patients presenting with menorrhagia were referred to hematology clinics by second level referral centers due to suspected bleeding disorders. The researcher filled out a form including demographic characteristics of the patients, bleedings other than menorrhagia, familial bleeding history, and characteristics of the menorrhagia during a face-to-face interview with the patient. Pictorial blood assessment charts (PBAC) were also distributed for evaluation of blood loss during menstruation. Patients compared the degree of saturation of their sanitary pads and tampons with those depicted on the chart. Patients ticked the pictures throughout menstruation and wrote the number of the tampons and/or pads used every day. Lightly stained pads or tampons obtained a score of 1, moderately stained pads or tampons a score of 5, and soaked pads or tampons a score of 20. The total points were calculated by the researcher. A PBAC total score of greater than 100 was considered a blood loss of more than 80 mL [6].

To evaluate the quality of life during menstrual periods, patients were asked to rate 7 parameters of daily living on a scale of 0-10, with 0 meaning that menses “does not interfere” with that parameter and 10 meaning that menses “completely interferes”. The parameters were patients’ general activity, ability to work and attend school, family activities, and ability to enjoy life, sleep, mood, and overall quality of life. Total points were 0-35 for the mildly affected and 36-70 for the severely affected [3]. All the patients had pelvic ultrasonography in the gynecology outpatient clinic and women also had pelvic examinations to exclude gynecological pathologies. Patients with gynecological pathologies such as myoma uteri or a history of systemic disease such as diabetes mellitus and hypothyroidism were excluded from the study. 

Blood samples were obtained in the first 5 days of the menstrual cycle and oral contraceptives (OCs) and non-steroidal anti-inflammatory drugs were withdrawn at least 10 days before. Vacutainer tubes with citrate were used and samples reached the local laboratory within 15 min. Aggregation assays were performed within 3 h. Whole blood count, peripheral blood smear, blood group, ferritin, platelet function analyzer 100 (PFA-100), prothrombin time (PT), activated partial thromboplastin time (aPTT), thrombin time (TT), fibrinogen, von Willebrand factor antigen (VWF:Ag), VWF ristocetin cofactor (VWF:RCo), factor VIII (FVIII), and platelet aggregation assays were performed. Serum transaminases, urea, and creatinine assays were also performed, because uremia impairs platelet functions and liver disease impairs synthesis of the coagulation factors. Other rare coagulation factor assays were performed when PT and/or aPTT were prolonged. Whole blood count was studied with the Celldyn-3700© (Abbot); biochemistry testing with the Aeroset 70© (Abbot); serum ferritin level with the Modular E-170© (Roche); PFA-100 testing with equipment by Dade-Behring©; PT, aPTT, fibrinogen, thrombin time, and VWF:Ag with Star© (Diagnostica Stago); and platelet aggregation testing and VWF:RCo with Chrono-Log Lumiaggregometry. Collagen, ristocetin, adenosine diphosphate (ADP), and epinephrine were used as agonists. The concentrations of agonists were: collagen, 0.5 mg/mL; ristocetin, 1.5 mg/mL; ADP, 1.0 µM/L; and epinephrine, 1.0 µM/L. Collagen and ADP cartridges were used for PFA-100. 

High PBAC score and/or duration of menses greater than or equal to 7 days with a sensation of flooding or bleeding through a tampon or pad in ≤2 h, history of treatment of anemia, clots greater than 2.5 cm in diameter, family history of diagnosed bleeding disorder, history of excessive bleeding with tooth extraction, delivery or miscarriage or surgery, history of spontaneous nosebleed that persisted for 10 min or needed medical attention, spontaneous gum bleeds, prolonged bleeding from minor wounds, easy bruising with minimal trauma, or similar bleeding symptoms in the family were regarded as clinical factors supporting existence of a bleeding disorder. The following criteria were used for diagnosis of bleeding disorders. Criteria for diagnosis of Glanzmann’s thrombasthenia (GT) were normal PT, aPTT, platelet count, and morphology; no curve with ADP, epinephrine, or collagen; and normal curve with ristocetin in platelet aggregation by lumiaggregometry. Criteria for diagnosis of Bernard-Soulier syndrome (BSS) were low platelet count, large platelets and normal aggregation with physiologic agonists, and no curve with ristocetin. For laboratory diagnosis of VWD, VWF:Ag, VWF:Rco, and FVIII coagulant assays were used. The normal value for these parameters was 50-200 IU/dL. VWF:Ag levels of 30 IU/dL or lower are required for the definite diagnosis of inherited VWD (especially type 1). VWF:Ag is less than 50 IU/dL in most patients with type 2A, 2B, or 2M VWD. VWF:Rco is less than 30 IU/dL in types 1 and 2. Levels of 30 to 50 IU/dL are classified as low VWD. In patients with blood group O, VWF:Ag is 25% lower. In type 1, both VWF:Ag and VWF:Rco are low and VWF:Rco/VWF:Ag is >0.5-0.7. In types 2A, 2B, and 2M, the VWF:Rco level is decreased more than VWF:Ag (VWF:Rco/VWF:Ag is <0.5-0.7). In types 1 and 2, the FVIII level may be low or normal. In type 2N, VWF:Ag and VWF:Rco may be normal or decreased. In type 3, VWF:Ag and VWF:Rco are absent (<3). In type 2N and type 3, FVIII is <30 IU/dL and <10 IU/dL, respectively [7]. Diagnostic criteria for immune thrombocytopenic purpura (ITP) was platelet count of <150,000/mm3 and normal or increased number of megakaryocytes and/or megakaryoblasts on marrow smear. 

After blood samples were obtained, iron salts, hormones (estrogen, progesterone, OCs), tranexamic acid, platelet concentrates, steroids, or fresh frozen plasma were administered regarding the underlying bleeding disorder. Hormonal therapies were prescribed by the consulting gynecologists of the outpatient clinic.

Statistical analysis was performed using SPSS 16. Descriptive statistics, chi-square test, and Fisher’s exact test were used. P<0.05 was considered statistically significant.

## RESULTS

During the study, 75 patients presented with menorrhagia, but 60 of them completed the study. Fifteen of the patients refused to come for platelet aggregation assays. The mean age of the patients was 20.68±10.34 (range: 10-48) years, and 65% (n=39) were younger than 18 years. Clinical and laboratory characteristics of the 75 enrolled patients are summarized in Table 1. In 20% (n=12) of the patients that completed the study, a bleeding disorder was detected (1 case of type 3 VWD, 2 cases of low VWF:Ag, 1 case of probable VWD, 3 of BSS, 2 of GT, 2 of ITP, and 1 of congenital factor VII deficiency). History and laboratory characteristics of the patients with congenital bleeding disorders are shown in Table 2. 

In patients with bleeding disorders, a history of mucosal bleeding, hypoferritinemia, and prolonged closure time were significantly more frequent (p<0.05). High PBAC score (>100), presence of anemia or severe anemia, mean hemoglobin, ovulation pain, menorrhagia beginning at menarche, and quality of life were not different between patients with and without bleeding disorders (Table 3). There was no difference between patients with and without congenital bleeding disorders in familial bleeding history, parental consanguinity, and high PBAC score. There was a positive correlation between PBAC score and anemia. 

In the medical history of the patients, the administered drugs due to menorrhagia were iron salts, tranexamic acid, combined OCs, and progesterone. Red blood cell transfusion was performed in 16 (26.6%) of the patients during follow-up. Oral iron salts, OCs, high-dose estrogen, tranexamic acid, apheresis platelets, packed red cells, and fresh frozen plasma were administered to the patients. Patients with ITP had pulse steroids for 3 days. The only surgical procedure was splenectomy in a patient with chronic ITP. There was partial response to the splenectomy (platelet count: 50,000-80,000/mm3), but this decreased menstrual blood loss. Patients were followed for 1 year with visits or phone calls. At the end of the first year of menarche in 18 (46%) of the adolescents, menorrhagia subsided spontaneously and was diagnosed as dysfunctional bleeding. All patients benefited from medical treatment. 

## DISCUSSION

At least 5%-10% of women of reproductive age will seek medical attention for menorrhagia [8]; however, an underlying etiology is identified in only 50% of cases [2]. The most common endocrinological cause of heavy menstrual bleeding in adolescent girls is anovulatory dysfunctional uterine bleeding owing to the immaturity of the hypothalamic–pituitary–ovarian axis [9]. In the present study, in nearly half of the adolescents, menorrhagia subsided spontaneously, probably with maturation of this axis, and no bleeding disorder could be detected.

It is clear that menorrhagia impairs quality of life in women. In the present study, 73.84% of women reported poor quality of life. In a study by Kouides et al., 46% of the patients with type 1 VWD reported losing time from work or school in the past 12 months because of menorrhagia. The median time lost was 4 (range: 1-24) days [3].

In a recent study from Sweden enrolling 152 women with idiopathic menorrhagia and 56 healthy, regularly menstruating women, a strong association was found between idiopathic menorrhagia and family history of heavy menstrual bleeding (r=0.68). The authors suggested that familial menorrhagia must be due to a hereditary trait [10]. In the present study, 39.58% of the patients without bleeding disorder had a family history of menorrhagia.

In this single center study, a bleeding disorder (VWD, other platelet function defects, or ITP) was identified in 20% of the patients, similar to the findings of James’ study [11]. A review of 11 studies conducted internationally found 13% (range: 11%-15.6%; 131 of 988 patients) of women with menorrhagia to have VWD [12]. In these studies, testing for other bleeding disorders of this population was not extensive, and only one study involved tests of platelet function beyond performance of a bleeding time test [13]. In a study performed in 6 centers in the United States, among 232 women with PBAC scores of >100, a laboratory abnormality was found in 73.3%, including both white (68.1%) and black (91.9%) subjects; 6.0% had VWD, 56.0% had abnormal platelet aggregation tests, 4.7% had a non-VWD coagulation defect, and 6.5% had an abnormal PFA only. Platelet aggregation was reduced in 58.9% of the subjects, with multiple agonists in 28.6%, a single agonist in 6.1%, and ristocetin alone in 4.2%. Laboratory abnormalities of hemostasis, especially platelet function defects, were common, but the clinical significance of these abnormalities was uncertain. This study also screened rare coagulation defects and compared factor levels of the patients with those of the control subjects. Twenty-three subjects had non-VWD coagulation defects (deficiencies of factors II, V, VII, XI, and XIII and fibrinogen, plasminogen activator inhibitor-1, and alpha-2-antiplasmin). Levels of these factors were above 50 IU/dL but slightly below the reference range, which may have limited the clinical significance [14]. Levels of factors V, VII, and X above 15%-20% and factor II above 20%-30% are generally considered adequate for hemostasis and thought not to be associated with significant bleeding. We did not study rare factor deficiencies routinely but measured the FVII level as 20 IU/dL in a patient with prolonged PT. According to some guidelines, in daily practice, initial tests for bleeding disorders should rule out more common causes of bleeding. These tests include whole blood counts, aPTT, PT, and possibly fibrinogen level or thrombin time. Patients with isolated prolonged PTT or with normal PTT, PT, platelet count, and fibrinogen level in the presence of bleeding signs or symptoms should receive VWF:Ag, VWF:RCo, and factor VIII assays to test for VWD [7].

 The diagnosis of VWD is complex and the diagnosis of mild forms can be difficult. Persons with levels of 30 to 50 IU/dL may need agents to increase VWF levels during invasive procedures or childbirth. Therefore, it is important to correlate severity and cause of bleeding with results of laboratory tests [7]. The levels of VWF and clotting factor VIII may vary depending on multiple factors, including age, race, genetic factors, blood type, stress, inflammation, hormones, and sample processing. When test results are low-normal or minimally decreased, the tests should be repeated if bleeding symptoms and family history suggest VWD [12]. In our series, patient 7 had prolonged aPTT and very low VWF antigen, VWF:RCo, and FVIII levels. Patient 8 had normal VWF:Ag and VWF:RCo levels (55 and 61 IU/dL), but she had familial history of menorrhagia, epistaxis, and a pathological curve with ristocetin in the platelet aggregation study. Diagnosis of VWD was not confirmed but the patient’s compliance was poor for repeating laboratory assays. Patients 6 and 9 had low VWF levels that were between 30 and 50 U/dL. 

Menorrhagia has been reported to be present in 51% of women with BSS and 13%-98% of women with GT [15,16,17]. In patients with GT and BSS, severe nose and gum bleeds in childhood were very striking, but the symptoms were not recognized by physicians and the patients were not referred to hematologists. These features were as important in the history of these patients as consanguineous marriage of the parents and bleeding history of the siblings. These defects show autosomal recessive inheritance. Only one patient with GT reported that her brothers had the same disease. Even in the United States, it was reported that there was a median of 16 years (0-39 years) between the first bleeding symptom and recognition of VWD in women. The average age at onset of bleeding symptoms was 6 years and at diagnosis of VWD was 23 years [18]. History of bleeding in surgery is important in bleeding disorders but this may be especially unhelpful in adolescents, because they might not yet have been exposed to such hemostatic challenges. 

The PFA-100 is easy to perform and a quick test, but is not the gold standard compared to lumiaggregometry. In our study, the number of the patients with bleeding disorders was too low to make a conclusion about the sensitivity and specificity of screening with PFA-100, although closure times seemed longer in patients with bleeding disorders. Eighty-one women with a physicians’ diagnosis of menorrhagia underwent PFA-100 and bleeding time tests to evaluate their effectiveness as screening tools for VWD and platelet dysfunction. Data showed that PFA-100 had a sensitivity of 80%, specificity of 89%, positive predictive value (PPV) of 33%, negative predictive value (NPV) of 98%, and efficiency 88% for VWD. For platelet aggregation defects, the PFA-100 closure time had a sensitivity of 23%, specificity 92%, PPV of 75%, NPV of 52%, and efficiency of 55%. Neither the PFA-100 nor the bleeding time tests are effective for purposes of classifying women for standard platelet aggregometry testing in women presenting with menorrhagia [13]. 

In a study by Kadir et al., menorrhagia since menarche was noted in 11 (8.9%) of 123 women without a bleeding disorder compared with 13 (65%) of 20 women with VWD (p=0.001) and 4 (66.7%) of 6 women with FXI deficiency (p<0.001) [19]. Although in our study, in patients with a bleeding disorder, menorrhagia since menarche was more frequent compared to the other patients (83.3% versus 66.7%), this difference was not significant (p=0.31). However, there were adolescents in our group and adolescents with dysfunctional menorrhagia had menorrhagia since menarche. 

It is clear that in the absence of iron-deficiency anemia, heavy menstrual bleeding is a subjective complaint and up to 50% of women describing menorrhagia will have a measured monthly blood loss within normal limits [20]. The PBAC score is useful in showing the severity of menorrhagia. Our study also confirmed that PBAC scores of >100 showed good correlation with anemia (p=0.05). 

Medical treatments including hormones, antifibrinolytics, and iron salts are useful in the management of patients without gynecological pathology regardless of the etiology. The concentrated spray form of desmopressin is not available in Turkey and the parenteral form can be used only in inpatient settings. Consequently, we did not try desmopressin (DDAVP) in patients with VWD. We used oral tranexamic acid in all patients and it reduced menstrual blood loss. Oral tranexamic acid (20-25 mg/kg every 8 h; generally a 1000-mg dose) for 5-7 days of the menstrual cycle was our practice. In some studies, a reduction in menstrual blood loss by 50% was shown [21]. It can be used in combination with OCs. In a recent study, tranexamic acid (1.3 g per os 3 times daily) showed a favorable safety profile, supporting its use as a therapy for cyclic heavy menstrual bleeding [22]. When OCs were compared with DDAVP nasal spray in managing menorrhagia in adolescents with type 1 VWD, they showed equivalent effectiveness. In DDAVP treatment, there were severe headaches and flushing [23]. Replacement with iron salts is also essential in the management of these women. It was reported that in some women with menorrhagia, iron deficiency impairs platelet aggregation, and this is reversed by iron replacement [24]. Adolescents were sexually inactive and unwilling to use OCs, and they generally used them for only 3 months. 

Unexplained hemorrhage often triggers surgical interventions such as endometrial ablation and/or hysterectomy. In our study, only one surgical intervention, splenectomy for chronic ITP, was performed. In adolescents and women of child-bearing age, preservation of fertility is essential. 

The limitations of this study were the low number of patients and the lack of routine screening of rare coagulation defects (II, V, VII, X, XI, XII, XIII) when PT, PTT, and TT were normal. FIX carriage was also not screened when there was no history of hemophilia B in the family. Multimer analysis of VWF was also not available. In addition, some assays were missing in some patients due to subjects’ incompliance to the study. We tried to compare some characteristics of the patients with and without underlying bleeding disorders to find predictive factors for underlying bleeding disorders. However, the statistical power was low due to patient numbers in the groups. 

In conclusion, screening of subjects with menorrhagia for underlying bleeding disorders is laborious and not all coagulation assays are available in many routine laboratories. In patients with menorrhagia, at least complete blood count, peripheral smear, aPTT, PT, VWF:Ag, VWF:RCo, and fibrinogen assays must be performed. When there is a history of nose and gum bleeding, platelet function assay by lumiaggregometer must also be performed. In patients with a history of surgical bleeding, screening of rare coagulation deficiencies is also required. In nearly 50% of adolescents, menorrhagia subsides with the maturation of the hypothalamic–pituitary–ovarian axis. Detailed coagulation assays can be postponed in adolescents if bleeding history other than menorrhagia and/or family history of bleeding and/or parental consanguinity is absent. All subjects with menorrhagia must consult with gynecologists and hematologists. In subjects with bleeding disorders, future follow-up for management of pregnancy and delivery is essential for the safety of the mother and offspring.

## ACKNOWLEDGMENT

Thanks to laboratory technician İlknur Çağlar for her meticulous work in all of the coagulation assays. Thanks also to the physicians that referred patients to us and performed gynecological evaluations. The Kocaeli University Research Foundation sponsored the study. There was no conflict of interest in this study. 

## Figures and Tables

**Table 1:**
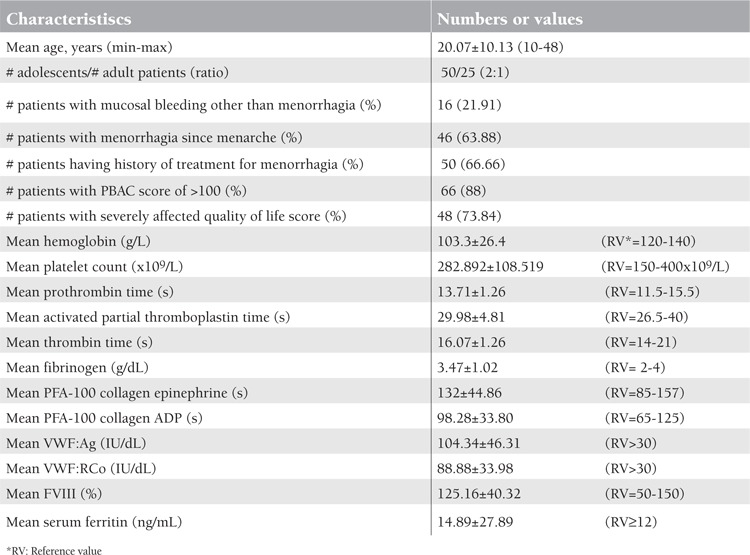
Clinical and laboratory characteristics of the patients with menorrhagia (n=75).

**Table 2:**
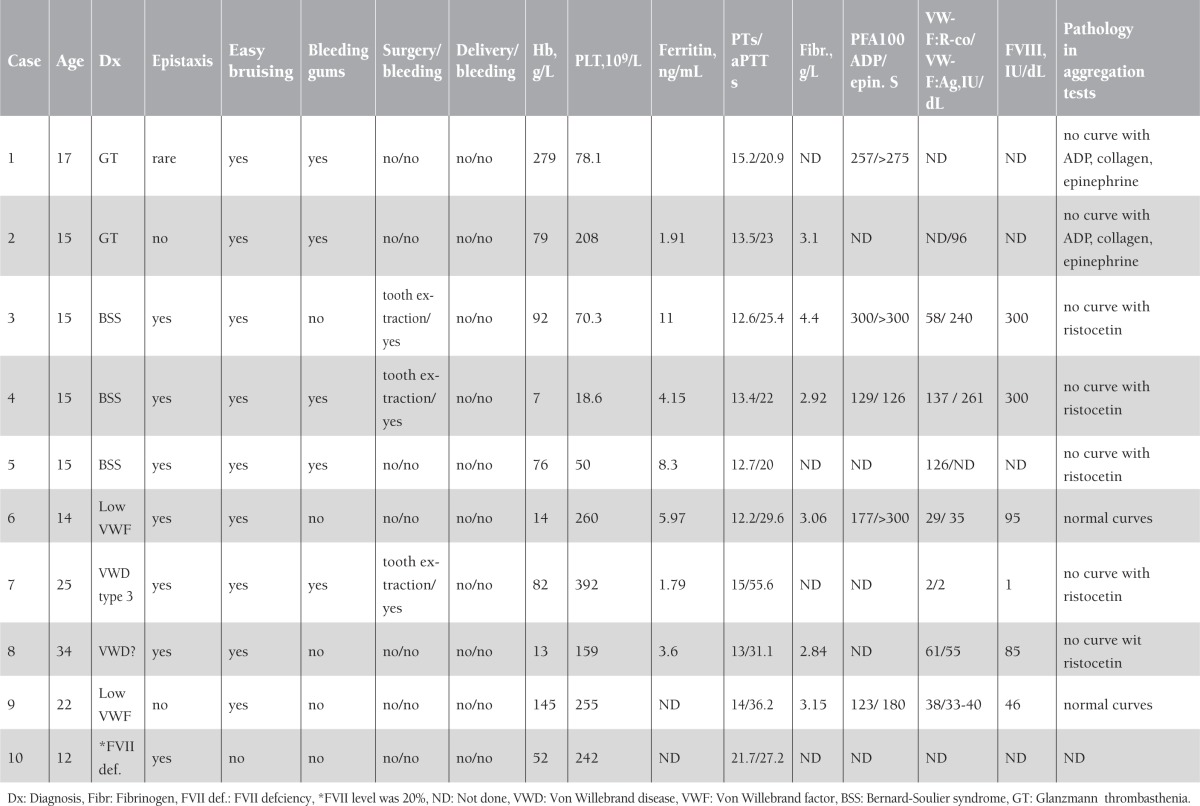
Bleeding history and laboratory characteristics of the patients with congenital clotting defects.

**Table 3:**
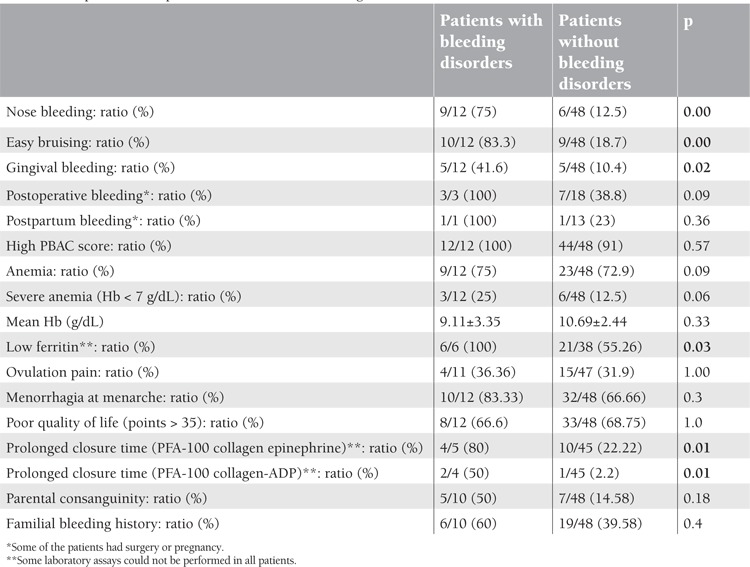
Comparison of the patients with and without bleeding disorders (n = 60)
